# Qualitative study on public health nurses’ experience and assessment of nutritional and physical activity counseling of women with gestational diabetes

**DOI:** 10.18332/ejm/127123

**Published:** 2020-09-22

**Authors:** Maiju Issakainen, Ursula Schwab, Reeta Lamminpää

**Affiliations:** 1 Karelia University of Applied Sciences, Joensuu, Finland; 2 School of Medicine, Institute of Public Health and Clinical Nutrition, University of Eastern Finland, Kuopio, Finland; 3 Department of Medicine, Endocrinology and Clinical Nutrition, Kuopio University Hospital, Kuopio, Finland; 4 Department of Nursing Science, University of Eastern Finland, Kuopio, Finland

**Keywords:** gestational diabetes, nutritional counseling, physical activity counseling, pregnancy, public health nurse

## Abstract

**INTRODUCTION:**

The number of pregnant women with gestational diabetes mellitus (GDM) has increased worldwide. GDM is a known risk factor for pregnant mothers and their fetuses that may increase various complications and health concerns. Nutrition and physical activity (PA) counseling during pregnancy can be crucial in supporting pregnant women to adopt healthier lifestyle practices and reducing these risks. This study describes public health nurses’ (PHNs) experiences of nutrition and PA counseling and their assessments on how to develop the counseling for pregnant women with GDM.

**METHODS:**

This is a descriptive qualitative study containing theme-interviews of 11 PHNs working in an antenatal maternity care setting. The data were analyzed using inductive content analysis.

**RESULTS:**

Five main themes were identified related to PHNs’ experiences and assessment of nutrition and PA counseling for pregnant women with GDM: competency of nutrition and PA counseling, challenges of counseling, positive experiences of counseling, printed material, and counseling practices. PHNs considered nutrition and PA counseling both challenging and rewarding. There was lack of knowledge and skills to provide proper counseling and adequate material to support versatile counseling.

**CONCLUSIONS:**

Material related to nutrition and PA counseling should be updated and standardized. PHNs need further training to improve knowledge in the area of diet and exercise.

## INTRODUCTION

Gestational diabetes mellitus (GDM) is abnormal glucose tolerance that develops or is diagnosed during pregnancy^1^. GDM is related to adverse perinatal outcomes for pregnant women and their newborns including preeclampsia, c-sections, and fetal macrosomia^2^. Children born to mothers who have GDM also face increased risks of obesity and metabolic syndrome during childhood^3,4^. Therefore, GDM prevention is important to reduce these risks.

In Finland, nearly all pregnant women (99.9%) regularly attend antenatal visits at maternity clinics. Public health nurses’ (PHNs) primary responsibility is to monitor pregnancy, provide guidance and advice on different aspects of a healthy lifestyle during pregnancy, such as balanced nutrition and physical activity (PA)^5^. Antenatal counseling at maternity clinics requires wide knowledge on variety of areas and knowledge in specific situations such as pregnancyrelated diagnosis of the woman, which makes the counseling a demanding process. In Finland, around 19% of pregnant women were diagnosed with GDM in 2018^6^. Regarding pregnant women with GDM, the aim of antenatal counseling is to focus on a healthy lifestyle as a whole. The overall aim is to minimize both the mother’s and baby’s chances of developing further morbidity later in life^3,7^. Internationally, there are a number of guidelines considering prevention and management of GDM indicating the importance of a healthy diet and exercise^8-10^. However, they are not always comprehensive or similar in recommended practices, and do not address the possible barriers to the implementation of the recommendations^9^.

It is known that counseling during pregnancy can change a pregnant woman’s lifestyle into incorporating healthier daily practices^11-14^. Lifestyle changes, including healthy diet and exercise, can positively affect the prevention of GDM or taking care of the existing disease^11,15-18^. Physical activity can act as a non-invasive option for preventing and managing GDM^19^.

Pregnant women these days are actively seeking information online related to, for example, dietary recommendations^20^, but the necessary skills to accurately evaluate the information is lacking^21^. In a study by Wennberg et al.^20^ it was shown that midwives viewed themselves as authorities possessing expert knowledge of antenatal care, but with neither adequate skills in nutrition nor competence in situations concerning sensitive issues such as weight. There was uncertainty related to the impact of their counseling on pregnant women’s behavior and their authority was seen both ambiguous and questioned^20^. It has been stated that professionals working in maternity clinics must be more educated in dietary and health counseling^22^, and pregnant women need accurate information, more time allocated for nutrition counseling, and a tailored approach enabling an interactive counseling environment^21^.

To our knowledge, there are few studies describing PHNs’ or midwives’ experiences and assessment of nutrition and PA counseling for pregnant women diagnosed with GDM. As the incidence of GDM is constantly increasing^2^ and the sources and quality of nutrition and physical activity related information is varying, it is important to explore health professionals’ experiences of antenatal counseling, which plays a key role in preventing GDM, and its further complications, as well as supporting the already diagnosed women. Thus, this study describes PHNs’ experiences of nutrition and PA counseling and their assessments on how to develop the counseling for pregnant women with GDM.

## METHODS

The results presented in this paper are from a wider study that aimed to explore the experiences and assessments of PHNs on providing nutrition and physical activity counseling to women with GDM and their perceived competency in providing the counseling. We presented results concerning PHNs’ experiences of counseling and their assessments on how to develop the counseling for pregnant women with GDM.

In this study we adopted a descriptive qualitative approach that provides a comprehensive description of the participants’ experiences of the topic^23^. PHNs who participated in this study worked at the same antenatal maternal health care setting in a Finnish city. Participants were recruited between November and December 2018. The managing nurse forwarded a recruiting letter containing detailed information about the study and the manner of interview by email to all potential participants. The PHNs willing to participate contacted the principal researcher by email. We used convenience sampling because all participants had to possess prior experience in providing nutrition and PA counseling. Participation was based on voluntary decisions and an informed consent was signed before interviews. Inclusion criteria for potential participants were: a licensed PHN (or midwife) and an employee at the selected healthcare organization.

The interviews took place between December 2018 and January 2019 at the antenatal maternity clinic (breakroom or meeting room). The principal researcher collected anonymous demographic information from all participants over the interviews. The group interviews were conducted by the principal researcher. Participants were able to suggest a suitable time for the interview. The interviews took place during the participants’ working hours. The groups for the interviews were composed of the participants themselves, and the participants who were able to participate at the same interview session agreed on the time and place in small groups. The themes on which the interviews were based on were shared with the participants beforehand, enabling them to be well prepared. The interviews consisted of a total of four themes: the quality and content of nutrition and PA counseling, factors affecting pregnant women’s willingness to follow the nutrition and PA guidelines, PHNs’ experiences of nutrition and PA counseling for women with GDM, and PHNs’ assessments on how to develop nutrition and PA counseling. In this paper we review the last two. The interviews were audiotaped for analysis. Data saturation was reached during the interviews, indicating the sufficiency of the gained data^24^.

The analysis was conducted by the principal researcher. The audiotaped material was listened through and transcribed verbatim. The written data were analyzed through inductive content analysis proceeding from original expressions towards general concepts. The transcribed text was read through several times and the meaning units related to the study’s aim were identified^25^. The meaning unit was typically a whole sentence. The meaning units, related to a certain theme, were labeled and eventually categorized as main themes and sub-themes.

Ethics approval was not required by the target organization as the study did not concern patients. Permission to conduct the study was granted by the organization.

## RESULTS

A total of 11 PHNs participated in the study. There were 4 group-interview sessions with the number of participants between 2 to 4. On average, an interview lasted approximately 50 minutes, and varied between 38 to 59 minutes.

The participants were all female and the age varied between 27 to 61 years (mean 47 years). Total work experience as a PHN varied from 0.5 years to 30 years (mean 14 years). Four of them (36%) had received education related to dietary and exercise counseling during pregnancy.

Results were divided into two main themes according to the study’s aims and presented based on main themes and sub-themes. The sub-themes are described using the participants’ own words.

### PHNs’ experience of nutrition and physical activity counseling for women with GDM

The first interview theme, related to experience of nutrition and PA counseling for women with GDM, included guiding questions on PHNs’ perceived competency and experiences of nutrition and PA counseling. The results were divided into three main themes: competency of nutrition and physical activity counseling; challenges of counseling; and positive experiences of counseling (Table 1).

**Table 1:** Themes of PHNs’ experience of nutrition and PA counseling for women with GDM

*Main themes*	*Sub-themes*
Competence of nutrition and physical activity counseling	Skills of basic knowledge
	Skills of specific knowledge
	Skills of giving counseling
Challenges of counseling	Psychological factors
	Pregnant woman’s individual factors
	Environmental factors
	Inadequate resources
Public health nurses’ skills	Factors related to printed material
Positive experiences of counseling	Rewarding feelings Collaboration

### Competency of nutrition and PA counseling

In this theme, PHNs described their level of competency related to nutrition and PA counseling as good or moderate.

‘Or at least I assume that our basic knowledge is reasonable. At least we provide a lot of guidance.’

They felt that they were providing relatively a lot of counseling and possessed good basic knowledge to do so. However, the level of their knowledge and skills varied. PHNs reported that their specific knowledge related to nutrition and PA counseling for women with GDM was inadequate. In particular, dietary issues were challenging, and the information they received, for example in educational sessions, was cursory.

‘But it is not self-evident if you don’t understand to read the information yourself from somewhere, you don't find the appropriate studies or you don't get training on the subject.’

They believed that the skills and knowledge needed in dietary and exercise counseling were so broad that they lacked the explicit information required to provide proper counseling and know-how of responding to problematic situations. PHNs also described the need to develop their counseling skills to act in counseling sessions while considering their existing skills to be generally good.

Meeting with the women was relaxed and PHNs felt that ability to pay attention to psychological issues is part of their professional identity.

‘And then these psychological skills that you need, they come from somewhere else. Maybe it is the encountering which comes naturally. It is the central part of being a nurse.’

### Challenges of counseling

In this theme, the participants described counseling-related challenges. They considered GDM to be a sensitive topic for pregnant women with the diagnosis, making it difficult for them. Hence, they described the need to possess psychological skills to be able to pay attention to the women’s feelings.

‘And then psychological support comes along.’

Negative feelings directed from the women during counseling can also be challenging. The participants described some individual factors that affect women’s ability to receive counseling, such as being defensive towards the GDM diagnosis, and refusing to understand the consequences related to this condition. In such cases, counseling can be difficult according to the nurses’ perspective. They stated that when women underestimate the diagnosis, it becomes challenging for them to proceed with counseling issues.

‘When you're trying to explain why it is important for you do these changes and the woman is just resisting.’

Motivating the women towards lifestyle changes can sometimes be the most challenging part of the counseling. Women’s attitudes towards exercise may also be a barrier in receiving counseling from PHNs. Sometimes women’s personal situations in life can also limit their commitment to lifestyle changes, such as economic difficulties.

‘Sometimes you have to think about how you approach your customer and how do you pay attention to her. And at the same time, you have to remain aware of the importance of the issue and uphold your professionalism. And you must take the issue forward from there, taking it seriously. Sometimes it's difficult.’

Environmental factors, such as pregnant women’s spouses, can impact behavior. Since they do not participate in the counseling process with the women, they are completely out of reach.

‘You can't really control the closest people around these women.’

The nurses claimed, since counseling was a timeconsuming process, that the lack of time and adequate resources pose a challenge. Many different issues need to be addressed in an antenatal visit, and therefore, the time for nutrition and PA counseling can be limited. PHNs described their skills in proper counseling also as being insufficient. Sometimes they also felt helpless when there was too little that could be done to control the woman’s situation with GDM.

‘Sometimes you feel like you have to take into account so many things in most of the clinic visits, and it feels like there isn't enough time for one thing.’

The printed material related to nutrition and PA to support counseling was also seen as inadequate, and hence a challenge to counseling.

‘This incoherence is confusing and you would need some good printed material.’

### Positive experiences of counseling

Positive counseling experiences were related to successful counseling sessions with positive results when the pregnant woman’s glucose level was successfully balanced. Successful counseling was described as rewarding and the PHNs felt that they managed well.

*‘It is very rewarding when you see the result of your work. For example, when we could stabilize the client's fluctuating blood sugar levels’*.

Counseling was also described as interesting because it enabled more specific and in-depth advice to be provided independently to each pregnant woman. They also felt that their collaboration with the hospital was good and guidelines related to further care of pregnant women with GDM were clear.

‘The collaboration with the hospital has been helpful.’

### PHNs’ assessments on how to develop dietary and exercise counseling

The second interview theme related to developing nutrition and PA counseling for pregnant women with GDM included guiding questions on how the nutrition and PA counseling at antenatal maternity clinics should be developed. The results were divided into two main themes: printed material; and counseling practices (Table 2).

**Table 2:** Themes on developing nutrition and PA counseling for women with GDM

*Main themes*	*Sub-themes*
Printed material	Updating the printed material
	Developing new guidelines material
Counseling practices	Consistent counseling practices
	Increasing resources
	Increasing group-counseling
	Developing multi-professional collaboration
	Further training for public health nurses

### Printed material

The nurses stated that the existing material was inadequate and outdated and needed further development. The printed material needs to be updated according to the newest information and more material should be made available. There was also inconsistency between different materials and guidelines, and hence consistency is required to ensure nothing was left open to interpretation.

‘Explicit material with nothing left open to interpretations that you could hand out to all the women.’

Material, especially related to physical activity, was not adequately available to support women with GDM. On nutritional issues, they expressed the need for developing more specific guidelines, which they could hand out to the women.

‘I don't know if it's just a crazy idea, but I'm pretty sure some of my clients would have benefited if I would have been able to give them an example of the week's menu.’

### Counseling practices

Regarding development of counseling practices, the PHNs indicated that the counseling should be consistent and standardized, leading to more homogenous counseling and contradictions avoided.

‘They don't have to get the same counseling, because the approach is customer oriented, but they should all get the homogenous information and the same quality.’

Regarding increasing resources, more time is needed to be spent in counseling to enable nurses to utilize their skills in more varied ways.

Leaning more towards group counseling practices was also brought up. In group counseling sessions, pregnant women with GDM could meet and get to know one another and receive peer support in various situations. The content of these group sessions could be based on nutrition guidance and perhaps some exercise in groups, which could motivate the women in terms of adopting new healthier lifestyle practices.

‘I don't know how it would work if we had a group for women with gestational diabetes. We could then exercise together with mothers. So, we could have some exercise first and then there could be a nutrition information afterwards.’

Multi-professional collaboration should be increased with experts from different healthcare areas, such as physiotherapists, physical education instructors, and dieticians. They could be included in taking group counseling sessions where women with GDM could express their concerns and receive guidance regarding multiple aspects of health and healthy lifestyle. The multi-professional aspect was also described in the PHNs’ further training where experts from different healthcare areas could participate in the same training session and receive the same information that would help counseling to become more consistent in the future. The PHNs also described the need for common meetings with different healthcare professionals that would make collaboration easier. The most important factor that needed to be developed was further training in nutrition and physical activity-related issues. PHNs described the lack of training opportunities that especially focused on nutrition and PA.

‘Well maybe there could be regular education about nutrition and exercise. Then we would all stay on the same page.’

## DISCUSSION

This study aimed to describe PHNs’ experience and assessment of nutrition and PA counseling for pregnant women with GDM, about which little is known. The counseling was considered both challenging and rewarding. Challenges were described in situations related to the pregnant women’s individual factors, guidance material, and their own lack of competency in the areas of nutrition and physical activity.

Patient attitudes, time restrictions, the potential sensitivity of the topic at hand (e.g. GDM diagnosis and weight gain), social concerns, knowledge, and accessibility of resources, have been previously identified to be barriers to antenatal counseling on the topic^8^. It is important that the PHNs also derived positive and successful experiences from the counseling process as the need for counseling is increasing along with the growing number of women diagnosed with GDM^19^. These results are similar to the existing literature describing the lack of training for health professionals related to nutrition^26-31^. An inconsistency, between different nutrition and PA guidelines related to the suggested type of physical activity and the content of diet recommendations, was raised and also the pregnant women’s view that the counseling was both contradictory and confusing^21,32-34^. In a review by Zhang et al.^8^ it was shown that there was a great inconsistency in the guidelines on the management of GDM that should be addressed.

In addition to individual guidance, the idea of group counseling sessions was highlighted as they would offer scope for peer support for those women who would benefit from them, as well as effective counseling practices. Group counseling could also decrease the number of individual counseling sessions and enable different experts, such as nutritionists and physical education instructors, to be included in the same counseling session. This result is supported by previous studies that have described women encountering peer support as an important issue during pregnancy^35,36^.

PHNs encourage pregnant women with GDM to follow a physically active and nutritional lifestyle. Counseling in antenatal maternity clinics is comprehensive and therefore a demanding area for PHNs. This is because they are expected to be competent to guide pregnant women on a variety of areas related to a healthy lifestyle. To be effective, counseling should also take place several times and consist of, for example, nutritional recommendations5. Counseling in specific situations, like women with GDM, is even more demanding since PHNs need to possess expert knowledge on this specific area and skills to tailor nutritional guidance individually based on recommendations while also considering the sensitivity of this topic. The lack of resources is a challenge to effective counseling process and results in failure to motivate the women towards a healthier lifestyle, according to different health professionals responsible for counseling^37^. In a study that explored counseling related to gestational weight gain, including nutrition and exercise issues, revealed that adequate knowledge about the topic is an on-going challenge and there is a need for the development and evaluation of knowledge translation tools to effectively address the topic in antenatal care^38^. Therefore, it is important to update the printed material and have clear guidelines as well as more education on the topic with all professionals who work with this group of pregnant women, in order to support an effective counseling process.

### Limitations

The study’s weakness lies in its small number of participants working at the same maternal healthcare setting (n=11), which limits the generalizability of the results. However, the data were saturated after the third interview when the interviews no longer provided new information. Our results also confirmed those of previous studies. It is possible that individual interviews rather than group interviews could have provided even more in-depth information on the topic, as group interviews may affect participants’ willingness to speak out openly. However, despite the small number of participants, the study strengths include diverse information on the topic, which highlights the need for further development of the antenatal counseling related to nutrition and PA for women with GDM.

## CONCLUSIONS

PHNs have comprehensive skills and knowledge on different areas of pregnancy-related issues, including nutrition and PA recommendations. However, in specific situations, such as women with GDM, the level of knowledge should be more detailed and tailored individually. The challenges to successful counseling process due to a lack of adequate resources and information were also revealed. PHNs should be provided with more training on nutrition and physical activity guidance to be able to adequately counsel women with GDM. The nutrition and physical activity guidance material should be updated, and multi-professional cooperation should be increased to develop the nutrition and PA guidance provided to such women. In addition to individually tailored guidance, group counseling could be effective in multiple ways in supporting and motivating such women so that they adopt and maintain healthy lifestyle practices.
